# 晚期非小细胞肺癌免疫治疗进展

**DOI:** 10.3779/j.issn.1009-3419.2021.102.06

**Published:** 2021-02-20

**Authors:** 浩洋 李, 敬慧 王

**Affiliations:** 1 101149 北京，北京市结核病胸部肿瘤研究所，首都医科大学附属北京胸科医院肿瘤内科 Department of Medical Oncology, Beijing Tuberculosis and Thoracic Tumor Research Institute, Beijing Chest Hospital, Capital Medical University, Beijing 101149, China; 2 101149 北京，北京市结核病胸部肿瘤研究所，首都医科大学附属北京胸科医院肿瘤研究中心 Cancer Research Center, Beijing Tuberculosis and Thoracic Tumor Research Institute, Beijing Chest Hospital, Capital Medical University, Beijing 101149, China

**Keywords:** 肺肿瘤, 免疫检查点抑制剂, 免疫治疗, Lung neoplasms, Immune checkpoint inhibitors, Immunotherapy

## Abstract

近年来，以免疫检查点抑制剂为代表的免疫治疗显著改善了晚期肺癌患者的生存，改变了肺癌的治疗模式。本文围绕免疫治疗的机制、免疫治疗的重要临床试验、关键生物标志物以及免疫相关不良反应进行综述，介绍了近年来晚期非小细胞肺癌免疫治疗发展过程中取得的成就和面临的挑战，展望了晚期非小细胞肺癌免疫治疗的未来前景。

## Abstract (en)

Immunotherapy, in particular immune checkpoint inhibitors, has significantly improved the survival outcomes of advanced lung cancer patients and changed the treatment mode of lung cancer. In this article, we reviewed the mechanism of immunotherapy, the clinical trials that changed treatment guidelines, the important biomarkers, immune-related adverse events, and descripted the future of immunotherapy of advanced non-small cell lung cancer.

晚期非小细胞肺癌（non-small cell lung cancer, NSCLC）是肿瘤内科治疗的难点，随着免疫检查点抑制剂（immune checkpoint inhibitors, ICIs）的发展，免疫治疗越来越广泛的应用于临床，革命性地改变了晚期NSCLC患者的治疗模式。本文回顾了免疫治疗的机制，梳理了标志性的临床试验，介绍了重要的生物标志物和免疫相关不良反应，讨论了晚期NSCLC免疫治疗的发展前景。

## 1 免疫治疗机制

免疫系统的重要功能是识别和清除非己成分，包括外源性抗原和体内异常细胞。免疫系统清除肿瘤的根本手段是效应T细胞的杀伤作用，肺癌不断进化的主要目标是逃避适应性免疫应答^[1]^。肿瘤免疫逃逸的方式方法有很多，直接逃逸有自身的弱免疫原性、下调表达肿瘤抗原、封闭抗原、低表达主要组织相容复合体（major histocompatibility complex, MHC）分子、缺乏共刺激分子、凋亡制止等; 间接逃逸可通过肿瘤微环境中的调节性T细胞、髓源性抑制细胞、巨噬细胞和B细胞，以及多种肿瘤相关免疫抑制因子和相应的炎症反应进行^[2, 3]^。目前临床治疗取得成功的主要是通过药物阻断抑制性受体与T细胞的相互作用，避免T细胞功能障碍。T细胞激活所需的共刺激信号主要有两种，一种是MHC与T细胞受体（T cell receptor, TCR）结合，另一种是CD86/80与CD28结合，这一免疫应答过程中有两个重要的免疫检查点，分别是细胞程序性死亡受体1（programmed cell death protein 1, PD-1）/细胞程序性死亡配体1（programmed cell death ligand 1, PD-L1）和细胞毒性T淋巴细胞相关蛋白4（cytotoxic T-lymphocyte-associated protein 4, CTLA-4）^[4, 5]^。

PD-1最初被认为是一种调节细胞死亡的分子，现在被认为是一种关键的检查点抑制受体，在抗原介导的刺激后改变T细胞的功能^[6]^。PD-1是免疫球蛋白超家族B7的一种Ⅰ型跨膜受体，它包含免疫受体酪氨酸基抑制基序（immunoreceptor tyrosine-based inhibitory motif, ITIM）和免疫受体酪氨酸基开关基序（immune receptor tyrosinyl switch motif, ITSM），在胸腺细胞、髓细胞、自然杀伤（natural killer, NK）细胞，尤其是生发中心相关T细胞、B细胞与肿瘤浸润淋巴细胞（tumor infiltrating lymphocytes, TILs）上表达^[7]^。PD-1的配体是PD-L1（B7-H1）和PD-L2（B7-DC），当它们与PD-1结合后，ITIM和ITSM被磷酸化，然后招募含蛋白酪氨酸磷酸酶2（SH2 domain-containing protein-tyrosine phosphatase-2, SHP2）的Src同源区2域，磷酸化磷脂酰肌醇3激酶（phosphatidylinositol 3-kinase, PI3K），传递抑制信号，最终抑制细胞因子的产生和T细胞的活化^[8, 9]^。T细胞、B细胞、巨噬细胞、内皮细胞和恶性细胞都能表达PD-L1。但是PD-L2在肿瘤细胞和巨噬细胞的表达较少。由于肿瘤基因激活或肿瘤抑制通路破坏，肿瘤细胞可以内源性诱导PD-L1表达，此外，一些促炎症因子也能够使PD-L1表达上调，如干扰素-γ（interferon-γ, IFN-γ）^[10, 11]^。多种研究表明，肿瘤微环境诱导PD-1/PD-L1通路会抑制肿瘤免疫反应，从而加速肿瘤的进展和转移。

细胞毒性T细胞相关蛋白-4（cytotoxic T lymphocyte associate protein-4, CTLA-4）又名CD152，是一种白细胞分化抗原，表达于活化的T细胞表面，与CD28同源^[12]^。APC通过MHC与T细胞表面受体结合同时还需要B7家族配体（CD80/CD86）与CD28结合的第二信号参与，才能激活T细胞。CTLA-4与B7家族配体亲和力强于CD28，抑制免疫反应。CTLA-4还可以下调APC上CD86或CD80的表达，或直接将CD86从APC表面去除，抑制CD28传递刺激信号^[13]^。CTLA-4激活使ITIM和SHP2或PP2A磷酸化，抑制肿瘤免疫^[9]^。

基于以上理论依据研制的PD-L1/PD-1抑制剂和CTLA-4抑制剂可以阻断抑制信号，诱导T细胞激活和增殖，恢复晚期癌症患者对肿瘤细胞的免疫杀伤能力。美国食品药品监督管理局（Food and Drug Administration, FDA）批准的可用于NSCLC治疗的PD-1药物主要有：纳武利尤单抗（Nivolumab）、帕博利珠单抗（Pembrolizumab）; PD-L1药物主要有：阿特珠单抗（Atezolizumab）、德瓦鲁单抗（Durvalumab）; CTLA-4药物主要有：伊匹单抗（Ipilimumab）和替西木单抗（Tremelimumab）。

## 2 免疫检查点抑制剂的临床研究

### 2.1 早期研究

2010年Brahmer等^[14]^最早发表了PD-1抑制剂治疗晚期实体肿瘤患者的研究结果。这是一项Ⅰ期临床研究，涉及包括NSCLC在内的多瘤种，共39例，这项研究首次证实了NSCLC免疫治疗的可行性并表明PD-1抗体治疗具有良好的安全性和可耐受性。随后，Topalian等^[15]^扩大规模在296例患者中开展了PD-1抗体的临床试验，同年Brahmer等^[16]^也发表了包含207例患者的PD-L1抗体临床试验的报告。这些研究进一步证明了PD-1/PD-L1阻断剂在NSCLC、黑色素瘤、结直肠癌、肾细胞癌、卵巢癌、胰腺癌、胃癌、乳腺癌中鳞癌和非鳞癌类型中具有持久的抗肿瘤作用。Topalian等^[15]^的研究还发现，17例PD-L1阴性肿瘤患者治疗无效，25例PD-L1阳性肿瘤患者中，9例（36%）有效，由此提出肿瘤细胞PD-L1表达水平可能是潜在的生物标志物。

### 2.2 免疫单药治疗

对于缺乏突变基因的NSCLC患者，标准一线治疗是含铂双药化疗联合或不联合抗血管药物。随着免疫治疗的发展，多项Ⅲ期临床研究证实了ICIs单药疗效优于化疗（表 1）。KEYNOTE-024和KEYNOTE-042均为帕博利珠单抗治疗与标准含铂化疗对于初治晚期NSCLC的疗效对照研究。KEYNOTE-024^[17, 18]^纳入患者的PD-L1肿瘤比例评分（tumor proportion score, TPS）≥50%，与化疗组相比，帕博利珠单抗治疗组客观缓解率（objective response rate, ORR）更高（44.8% *vs* 27.8%）; 无进展生存时间（progression free survival, PFS）更长（10.3个月 *vs* 6.0个月; HR=0.50;95%CI: 0.37-0.68;*P* < 0.001）; 总生存时间（overall survival, OS）更长（30.0个月 *vs* 14.2个月; HR=0.63;95%CI：0.47-0.86，*P*=0.002）; 治疗相关不良反应发生率也更低（73.4% *vs* 90%）。KEYNOTE0-24的试验结果使得FDA批准帕博利珠单抗治疗作为PD-L1表达阳性（TPS≥50%）驱动基因阴性NSCLC患者的一线治疗方案。KEYNOTE-042^[19]^纳入患者的PD-L1 TPS≥1%，研究发现帕博利珠单抗治疗组比化疗组的OS更长（16.7个月 *vs* 12.1个月; HR=0.85;95%CI：0.71-0.93，*P*=0.001, 8）; 分层分析结果表明，PD-L1 TPS≥50%的OS获益最显著（20个月 *vs* 12.2个月; HR=0.69;95%CI：0.56-0.85;*P*=0.000, 3）而TPS≥1%-49%的OS获益无显著差异。另外，帕博利珠单抗治疗组三级以上治疗相关不良事件发生率更低（18% *vs* 41%）。KEYNOTE-010^[20]^试验随机分配PD-L1 TPS≥1%患者进行2 mg/kg和10 mg/kg帕博利珠单抗治疗与多西他赛化疗对比，不同剂量帕博利珠单抗治疗组的OS（10.4个月 *vs* 12.7个月 *vs* 8.5个月）均优于化疗。

**1:** 单药免疫治疗临床研究 Clinical trials of ICI monotherapy

Study	Phase	Histology, PD-L1	Line	Study design	Key findings	HR (95%CI)
KEYNOTE-024^[18]^	Ⅲ	NSCLC, PD-L1 TPS≥50%	First-line	Pembrolizumab *vs* platinum-based chemotherapy	mOS: 30.0 mon *vs* 14.2 mon	0.63 (0.47-0.86)
KEYNOTE-042^[19]^	Ⅲ	NSCLC, PD-L1 TPS≥1%	First-line	Pembrolizumab *vs* platinum-based chemotherapy	mOS: 16.7 mon *vs* 12.1 mon	0.85 (0.71-0.93)
KEYNOTE-010^[20]^	Ⅱ/Ⅲ	NSCLC, PD-L1 TPS≥1%	Second-line	Pembrolizumab 2 mg/kg or10 mg/kg *vs* docetaxel	mOS: 10.4 mon *vs* 8.5 mon (2 mg/kg); mOS: 12.7 mon *vs* 8.5 mon (10 mg/kg)	2 mg/kg: 0.7110 mg/kg: 0.61
CheckMate026^[21]^	Ⅲ	NSCLC, PD-L1 TPS≥1%	First-line	Nivolumab *vs* platinum-based chemotherapy	mOS: 14.4 mon *vs* 13.2 mon	1.02 (0.80-1.30)
MYSTIC^[22]^	Ⅲ	NSCLC	First-line	Durvalumab *vs* valumab+Tremelimumab *vs* platinum-based chemotherapy	mOS: 16.3 mon *vs* 12.9 mon（D *vs* Chemo）mOS: 11.9 mon *vs* 12.9 mon（D+T *vs* Chemo）	D *vs* Chemo 0.76 (0.56-1.02)D+T *vs* Chemo 0.85 (0.61-1.17)
CheckMate017^[23]^	Ⅲ	Squamous	Second-line	Nivolumab *vs* docetaxel	mOS: 9.2 mon *vs* 6.0 mon	0.62 (0.47-0.80)
CheckMate057^[24]^	Ⅲ	Nonsquamous	Second-line	Nivolumab *vs* docetaxel	mOS: 12.2 mon *vs* 9.4 mon	0.75 (0.63-0.91)
OAK^[25]^	Ⅲ	NSCLC	Second-line	*Atezolizumab vs* docetaxel	mOS: 13.8 mon *vs* 9.6 mon	0.73 (0.62-0.87)
PD-L1: programmed cell death ligand 1; ICIs: immune checkpoint inhibitors; TPS: tumor proportion score; NSCLC: non-small cell lung cancer; mOS: median overall survival; mPFS: median progression-free survival; Chemo: chemotherapy.						

然而，Ⅲ期临床试验CheckMate026^[21]^的结果并不理想，对于PD-L1 TPS≥5%的患者，纳武利尤单抗与铂类化疗的疗效对比，PFS（4.2个月 *vs* 5.9个月）和OS（14.4个月 *vs* 13.2个月）均无显著差异。MYSTIC试验比较了德瓦鲁单抗、德瓦鲁单抗联合替西木单抗分别与含铂双药化疗对于PD-L1 TPS≥25%患者的疗效，PFS和OS均无显著差异^[22]^。

CheckMate017^[23]^和CheckMate057^[24]^分别对接受二线治疗的鳞癌和非鳞癌NSCLC患者进行了纳武利尤单抗与多西他赛化疗的对照研究。CheckMate017研究结果表明纳武利尤单抗能够改善OS（9.2个月 *vs* 6个月; HR=0.59;95%CI：0.44-0.79;*P* < 0.001）和PFS（3.5个月 *vs* 2.8个月; HR=0.62），PD-L1表达不能预测疗效。CheckMate057研究结果表明武利尤单抗能够改善OS（12.2个月*vs* 9.4个月; HR=0.73;95%CI：0.59-0.89;*P* < 0.002），而且PD-L1表达与疗效获益相关。两项研究均发现纳武利尤单抗组的ORR比化疗组高（20% *vs* 9%; 19% *vs* 12%），治疗相关不良反应比化疗组低（58% *vs* 86%; 69% *vs* 88%）。OAK研究^[25]^纳入晚期NSCLC患者，不区分病理类型，不限定PD-L1表达，比较阿特珠单抗和多西他赛的治疗效果。研究表明，PD-L1高的组临床获益更好，但无论PD-L1表达如何，阿特珠单抗都能够提高OS（13.8个月 *vs* 9.6个月; HR=0.73;95%CI：0.62-0.87;*P*=0.000, 3），也能降低治疗相关不良反应的发生（15% *vs* 43%）。

总的来说，这些临床试验或多或少证实了ICIs单药治疗相对标准化疗在OS，ORR，不良反应方面更具优势。但是，临床试验也暴露出PD-L1检测方法不统一、判定阈值不一致、预测能力不完善等问题。目前，美国国立综合癌症网络（National Comprehensive Cancer Network, NCCN）指南只推荐ICIs单药作为PD-L1 TPS≥50%驱动基因阴性NSCLC患者的一线治疗方案。

### 2.3 免疫联合化疗

免疫联合化疗的理论基础在于化疗药物可以杀伤肿瘤细胞，增加肿瘤抗原释放，也可以抑制Treg细胞，激活DC细胞和NK细胞，诱导肿瘤细胞PD-L1表达^[26-28]^。免疫联合化疗的研究现已有多项临床试验结果（表 2）。KEYNOTE-189^[29]^研究是一项随机、对照、双盲设计的Ⅲ期临床试验，入组初治的表皮生长因子受体（epidermal growth factor receptor, *EGFR*）突变阴性或ALK阴性的晚期非鳞NSCLC患者，按2:1比例随机入组到帕博利珠单抗联合培美曲塞和铂类组或安慰剂联合培美曲塞和铂类组。研究结果证实帕博利珠联合化疗可以显著延长OS（22.0个月 *vs* 10.7个月; HR=0.57;95%CI：0.45-0.70）和PFS（9.0个月 *vs* 4.9个月; HR=0.48;95%CI：0.40-0.58;*P* < 0.001）。研究还发现帕博利珠单抗联合化疗组改善PFS和OS的效果，与PD-L1表达情况和是否发生肝、脑转移无关; 帕博利珠联合化疗组与单纯组的3级-5级不良反应发生率接近（71.9% *vs* 66.8%）^[30]^。KEYNOTE-407是在晚期肺鳞癌患者中比较帕博利珠单抗联合化疗与单纯化疗的随机Ⅲ期临床研究。研究结果表明，无论PD-L1表达如何，帕博利珠单抗联合化疗组可以改善PFS（6.4个月 *vs* 4.8个月; HR=0.56;95%CI：0.45-0.70）和OS（15.9个月 *vs* 11.3个月; HR=0.64;95%CI：0.49-0.85;*P* < 0.001）; 两组不良反应发生率接近^[31]^。同样比较晚期肺鳞癌免疫联合化疗与化疗疗效的Ⅲ期临床试验IMpower131，阿特珠单抗联合白蛋白紫杉醇卡铂组与白蛋白紫杉醇卡铂组相比，PFS有改善（6.3个月 *vs* 5.6个月; HR=0.715;95%CI：0.603-0.848;*P*=0.000, 1），OS无差异^[32]^。IMpower132对比了阿特珠单抗联合化疗与化疗在晚期非鳞NSCLC患者中的治疗效果，结果表明，无论PD-L1表达情况，阿特珠单抗联合组能够改善PFS（7.6个月 *vs* 5.2个月）^[33]^。

**2:** 免疫联合化疗临床研究 Clinical trials of ICI with chemotherapy

Study	Phase	Histology, PD-L1	Line	Study design	Key findings	HR (95%CI)
KEYNOTE-189^[30]^	Ⅲ	Nonsquamous	First-line	Carboplatin/cisplatin+ pemetrexed/pembrolizumab	12-mon OS: 69.2% *vs* 49.4%	0.49 (0.38-0.64)
KEYNOTE-407^[31]^	Ⅲ	Squamous	First-line	Carboplatin/paclitaxel or nab-paclitaxel/pembrolizumab	mOS: 15.9 mon *vs* 11.3 mon	0.64 (0.49-0.85)
IMpower131^[32]^	Ⅲ	Squamous	First-line	Carboplatin+ nab-paclitaxel/ atezolizumab	mPFS: 6.3 mon *vs* 5.6 mon	0.715 (0.603-0.848)
IMpower132^[33]^	Ⅲ	Nonsquamous	First-line	Carboplatin/cisplatin+ pemetrexed/atezolizumab	mPFS: 7.6 mon *vs* 5.2 mon	0.60 (0.49-0.73)
IMpower150^[34]^	Ⅲ	Nonsquamous, including EGFR/ALK+	First-line	Carboplatin/paclitaxel+ bevacizumab/atezolizumab	mOS: 19.2 mon *vs* 14.7 mon	0.78 (0.64-0.96)
EGFR: epidermal growth factor receptor; ALK: anaplastic lymphoma kinase.						

Impower150研究更加复杂，对晚期非鳞NSCLC设计了阿替利珠单抗+贝伐珠单抗+卡铂+紫杉醇（ABCP组）和阿替利珠单抗+卡铂+紫杉醇（ACP组）分别对比贝伐珠单抗+卡铂+紫杉醇（BCP组），研究者意在回答抗血管生成药物能否进一步提高免疫联合治疗的疗效，以及驱动基因阳性患者能否在免疫治疗中获益的问题。研究结果表明，ABCP组的PFS（8.3个月 *vs* 6.8个月）和OS（19.5个月 *vs* 14.7个月）均优于BCP组; 亚组分析发现，ABCP方案能够改善EGFR阳性患者OS（29.4个月 *vs* 18.1个月; HR=0.6;95%CI：0.31-1.14）; 无论患者PD-L1的表达状态如何，ABCP组较BCP组均有不同程度OS的改善^[34]^。

这些临床研究使得免疫联合化疗成为PD-L1 TPS≥1%的驱动基因阴性NSCLC患者的标准一线治疗。但是，对于PD-L1 TPS≥50%的患者选择单纯免疫治疗还是免疫联合化疗，如何筛选能够免疫治疗获益的PD-L1阴性患者以及如何让驱动基因阳性的患者通过免疫治疗获益，仍需要更多针对性的临床试验数据支持。

### 2.4 双免疫检查点抑制剂联用

CheckMate227是第一个评价纳武利尤单抗联合低剂量伊匹单抗在晚期NSCLC一线治疗疗效的临床试验。研究纳入未经化疗的晚期或复发性NSCLC患者，先按PD-L1表达分为TPS≥1%组和TPS < 1%组，然后再分别随机分为纳武利尤单抗、帕博利珠单抗+伊匹单抗、纳武利尤单抗+铂类双药化疗，铂类双药化疗四组^[35]^。由于CheckMate568^[36]^发现无论PD-L1表达水平如何，肿瘤突变负荷（tumor mutational burden, TMB）≥10 mut/Mb均与纳武利尤单抗联合伊匹单抗改善ORR和PFS有关，CheckMate227又按照这一标准增加了TMB分组。研究结果表明：PD-L1≥1%患者，帕博利珠单抗联合伊匹单抗组与化疗组相比，OS得到改善（17.1个月 *vs* 14.9个月; HR=0.79;95%CI：0.65-0.96;*P*=0.007），缓解持续时间也更长（23.2个月 *vs* 6.2个月）; 无论PD-L1表达如何，ORR与TMB呈正相关，而且TMB≥10 mut/Mb组的双免疫联合能改善PFS（7.2个月 *vs* 5.5个月; HR=0.58;95%CI：0.41-0.81，*P* < 0.001）; 无论TMB表达如何，双免疫联合能够改善OS; 双免疫联合组与化疗组3级以上不良反应发生率接近（32.8% *vs* 36.0%）^[35]^。

### 2.5 其他新型药物临床试验

除了PD-1/PD-L1和CTLA-4，基于其他免疫检查点研发的药物也在不断发展中。淋巴细胞活化基因3（lymphocyte-activation gene 3, LAG-3）与纤维介素蛋白1（fibrinogen like protein 1, FGL1）结合后可以抑制T细胞增殖及免疫活性^[37]^。LAG-3抗体Eftilagimod Alpha联合帕博利珠单抗治疗转移性NSCLC的Ⅱ期临床试验已有初步结果，可评估的17例患者中，9例（53%）部分缓解（partial response, PR），5例（29%）疾病稳定（stable disease, SD），ORR为53%，疾病控制率（disease control rate, DCR）达到82%;所有PD-L1表达亚组均有PR患者; 随访超过9个月后，仍有7例（41%）患者在治疗中，中位PFS未达到。最常见的不良反应（超过10%）是咳嗽（29%）、虚弱（24%）、食欲减退（18%）、呼吸困难（18%）、乏力（17%）、腹泻（15%）和恶心（12%）^[38]^。探索靶向LAG-3和PD-L1的四价双特异抗体MGD013在晚期实体瘤和血液系统恶性肿瘤中安全性和耐受性的Ⅰ期临床研究也在进行中^[39]^。

T细胞免疫球蛋白和ITIM结构域蛋白（T cell immunoglobulin and ITIM domains, TIGIT）是淋巴细胞上表达的抑制性免疫受体，作为PVR/nectin家族中重要的抑制分子，它与人类肿瘤和T细胞衰竭表型相关。抑制TIGIT可以增强抗肿瘤T细胞反应^[40]^。Johnson在2020年美国肿瘤学会年会（American Society of Clinical Oncology Annual Meeting, ASCO）上公布了TIGIT抗体Tiragolumab联合阿特珠单抗治疗局部晚期或转移性NSCLC患者的Ⅱ期临床试验（CITYSCAPE）的初步结果，与阿特珠单抗治疗组相比，联合治疗组ORR（37% *vs* 21%）和PFS（5.6个月 *vs* 3.9个月）有改善; 联合治疗PD-L1≥50%组的ORR高于PD-L1低表达组（66% *vs* 16%）; 联合治疗组不良反应发生率也更高（69% *vs* 47%）^[41]^。2020年的欧洲肿瘤学年会（European Society for Medical Oncology, ESMO）公布了另一种TIGIT抗体Vibostolimab对于初治晚期NSCLC安全性和耐受性试验的初步结果，79例患者中41例接受Vibostolimab单药治疗，38例接受Vibostolimab联合帕博利珠单抗治疗，两组患者的治疗相关不良反应发生率均超过65%，最常见的是瘙痒、疲劳、皮疹、关节痛以及食欲减退，有10例患者发生3级-4级不良反应，1例接受联合治疗的患者死于治疗相关肺炎，单药治疗组的ORR为7%，联合治疗组的ORR为5%^[42]^。

T细胞免疫球蛋白和粘蛋白结构域蛋白3（T cell immunoglobulin and mucin domain-3, TIM-3）在多种免疫细胞表达，与抑制T细胞功能相关。阻断TIM-3能够恢复T细胞功能，并增强PD-1/PD-L1阻断能力^[43]^。Ⅰ期临床试验^[44]^表明抗TIM-3药物LY3321367对于晚期实性肿瘤患者耐受性良好。唾液酸结合免疫球蛋白样凝集素-15（Siglec-15）在免疫抑制性M2巨噬细胞和包括肺癌在内的多种肿瘤中表达，它能够抑制T细胞功能^[45]^。一项Ⅰ期临床试验对49例后线免疫治疗耐药的晚期肿瘤患者使用Siglec-15抗体NC318药物，33%的患者疾病得到控制。参加试验的13例晚期NSCLC患者，有1例完全缓解（complete response, CR），1例PR，4例SD^[46]^。

另一类免疫治疗药物通过调节共刺激分子增强免疫微环境的抗肿瘤功能。诱导性共刺激分子（inducible co-stimulator, ICOS）的激动剂抗体GSK3359609能够选择性增强T细胞功能，在治疗头颈部鳞状细胞癌的Ⅰ期临床试验^[47]^中发现，接受GSK3359609联合帕博利珠单抗治疗的34例患者中，ORR为26%（4例CR，5例PR），DCR达到68%，中位PFS为5.6个月，治疗相关不良反应发生率为66%。T细胞的共刺激分子OX40能促进CD8^+^ T细胞活化增殖，并抑制Treg细胞，增强抗肿瘤免疫反应。OX40激动型抗体MEDI0562联合德瓦鲁单抗或替西木单抗的Ⅰ期临床试验评估了药物对于晚期实体肿瘤的安全性和临床疗效，全部57例患者总ORR为5.3%，DCR为30.8%，中位PFS为1.9个月，中位OS为11.9个月^[48]^。免疫细胞和非免疫细胞表达的CD73均可以促进肿瘤的免疫逃逸、发展和转移，靶向CD73的抗体或基因敲除CD73可以有效阻断肿瘤生长和转移，研究^[49, 50]^还发现了CD73独立于其腺苷酸催化活性的其他功能，如介导细胞黏连和迁移。抗CD73药物Oleclumab在转移性三阴乳腺癌的Ⅰ期/Ⅱ期临床试验表明，药物耐受性良好，24周疗效评价时，6例患者中有4例获得PR^[51]^。

白介素2（interleukin-2, IL-2）家族细胞因子也有新的治疗进展。IL-2被批准用于治疗肾细胞癌和黑色素瘤已有二十多年历史，但是其应用受限于毒副反应大、半衰期长以及对调节性T细胞的刺激。基于生物技术改造的IL-2变种药物NKTR214和ALKS4230能够避免刺激调节性T细胞，毒副反应更低，半衰期更长^[52, 53]^。NKTR214和ALKS4230联合PD-1药物治疗晚期实体瘤的几项临床试验正在进行中，并在黑色素瘤、卵巢癌、肾细胞癌治疗中初见成效^[54, 55]^。

## 3 生物标志物

针对非选择性人群，ICIs单药ORR为19%-22%，为使免疫治疗获益最大化，需要能够分层识别患者的预测性生物标志物。截止目前，免疫组化检测PD-L1表达是研究最多的也是唯一被批准的用于NSCLC的伴随诊断。最初，每种单药临床试验都会选择一种PD-L1抗体并给出各自的评价标准，导致不同临床试验的结果不一致。KEYNOTE-024研究^[17]^表明PD-L1≥50%的患者能够从帕博利珠单抗单药治疗获益，而CheckMate017研究^[23]^表明，PD-L1表达与疗效无明显关联。出现这样结果还与肿瘤PD-L1表达的空间异质性和时间异质性有关^[56, 57]^。目前，PD-L1免疫组化检测抗体主要有5种：22C3、28-8、SP263、SP142和73-10，分别在两个免疫组化平台Dako和Ventana进行检测。FDA批准了22C3（Dako）的PD-L1检测作为帕博利珠单抗的伴随诊断，28-8（Dako）和SP142（Ventana）则分别为纳武利尤单抗和阿特珠单抗的补充诊断。SP263（Ventana）被欧盟认证作为三种免疫抑制剂的补充诊断。为了验证PD-L1检测的一致性，国外做了Blueprint研究，结果表明22C3、28-8和SP263在肿瘤区染色一致性较好，SP142一致性较差; 研究还对比了不同细胞的染色效果，发现肿瘤细胞染色一致性好，免疫细胞染色一致性较差^[58, 59]^，由此，NSCLC主要采用22C3抗体和TPS评分标准。即便如此，PD-L1表达仍不完美，PD-L1高表达（TPS≥50%）的晚期NSCLC患者一线免疫治疗的ORR仅44%。

TMB是指每百万碱基中被检测出的，体细胞基因编码错误、碱基替换、基因插入或缺失错误的总数^[60]^。一项回顾性研究^[60]^发现，在接受PD-1或PD-L1抑制剂治疗的27个瘤种患者中，ORR与TMB水平呈正相关。CheckMate227的研究结果也发现在肺癌中TMB高的患者ORR和PFS有改善^[61]^。然而，KEYNOTE-021和KEYNOTE-189表明TMB与帕博利珠单抗联合化疗疗效无关。原因可能是化疗杀伤肿瘤细胞，释放肿瘤抗原，改变了肿瘤微环境，增强了ICIs的作用效果，也影响了治疗前TMB水平与联合治疗疗效的相关性。另外，进一步的肺癌治疗研究^[62, 63]^显示，高PD-L1表达与高TMB群人并不重合，两者并无相关性。TMB的局限性还在于方法本身，全外显子测序的复杂与昂贵以及难以确定的复杂阈值标准是最大的阻碍。随着技术的发展，血液肿瘤突变负荷（blood tumor mutational burden, bTMB）相关研究也在进行中。基于阿特珠单抗治疗的临床研究表明，bTMB和TMB阳性一致率为64%（95%CI: 54%-74%），阴性一致率为88%（95%CI: 83%-92%）^[64]^。

T细胞炎症基因表达谱（gene expression profile, GEP）也是一种有潜力的预测手段。Ayers等^[65]^使用接受帕博利珠单抗治疗患者的基线肿瘤mRNA分析GEP，发现T细胞炎症GEP包含与抗原呈递、趋化因子表达、细胞毒活性和适应性免疫抗性相关的干扰素-γ应答基因。IFN-γ是癌症和宿主细胞中PD-L1表达的关键驱动因素^[66]^。Cristescu等^[67]^研究发现接受帕博利珠单抗治疗的TMB和GEP双高表达的患者相较TMB或GEP表达低或双低的患者，PFS得到改善，提示TMB和GEP有联合预测能力。

其他生物标志物，包括免疫微环境、外周血细胞因子、肠道菌群等方向的研究也在进行中，新发现有见报道，但都缺乏大规模临床数据验证，距离临床应用还有一段距离。

## 4 免疫相关不良反应

免疫相关不良反应（immune-related adverse events, irAEs）是ICIs导致的炎性反应，它的发病机制还未被研究透彻，暂时认为与免疫稳态被破坏有关^[68]^。与化疗相比，免疫治疗的不良反应发生率更低，但是由于免疫药物作用机制，irAEs可以发生在全身几乎所有的系统和器官。irAEs好发于胃肠道、内分泌腺、皮肤、肝脏和肺，也可累及肌肉骨骼系统、神经系统、肾脏和心脏。一项回顾性荟萃分析表明：最常见的全身不良反应为疲劳（18.26%, 95%CI: 16.49%-20.11%）、瘙痒（10.61%, 95%CI: 9.46%-11.83%）和腹泻（9.47%, 95%CI: 8.43%-10.58%）; 最常见的3级或以上不良事件为乏力（0.89%, 95%CI: 0.69%-1.14%）、贫血（0.78%, 95%CI: 0.59%-1.02%）和转氨酶升高（0.75%, 95%CI: 0.56%-0.99%）^[69]^。

ICIs增强免疫系统活性，导致T细胞攻击机体的健康细胞，产生类似自身免疫病的症状，当累及重要器官如肺、心脏时会很严重。免疫相关性肺炎症状包括咳嗽、呼吸困难、喘息、胸痛和发热，影像学和病理表现为间质性肺炎^[70]^，严重的会危及生命; 其原因可能与PD-L2参与呼吸系统耐受作用有关^[71]^。免疫相关性心肌炎是一种少见但严重的irAE，世界卫生组织（World Health Organization, WHO）数据显示其死亡率高达36%-67%^[72]^; PD-1和PD-L1在人心肌细胞中均有表达，曾有研究^[73]^表明CTLA-4和PD-1缺失可引起自身免疫性心肌炎。irAEs可以发生在治疗过程中的任意时刻，甚至在治疗结束后，症状表现不一，轻重不一，美国国立综合癌症网络（National Comprehensive Cancer Network, NCCN）和ASCO已有应对irAEs的系统指南^[74, 75]^内容涵盖所有类型包括具体的药物治疗措施，国内的专家共识也在跟进，对待irAEs应当谨慎观察，早发现、早处理。

## 5 总结

经过多年发展，ICIs正在使越来越多的晚期NSCLC患者获益。单药免疫治疗，免疫联合化疗以及其他免疫联合治疗方案丰富了治疗手段，也给临床医生带来了新的挑战，首当其冲的是如何筛选获益人群; 伴随免疫治疗而来的irAEs以及耐药现象也需要更进一步的研究。不管是对于疗效、预后，还是毒副反应，共同的痛点是缺乏完善的生物标志物，就目前趋势来看，整合多种生物标志物，建立疗效预测模型是未来的发展方向。总之，免疫治疗的发展需要基础医学的进一步深入研究，也需要临床医生的经验和总结，共同的目标是扩大受益人群，提高诊疗水平，早日实现精准免疫治疗。
